# Inflammasome expression is higher in ovarian tumors than in normal ovary

**DOI:** 10.1371/journal.pone.0227081

**Published:** 2020-01-10

**Authors:** Judith Luborsky, Animesh Barua, Seara Edassery, Janice M. Bahr, Seby L. Edassery

**Affiliations:** 1 Department of Pharmacology, Rush University Medical Center, Chicago, Illinois, United States of America; 2 Department of Obstetrics & Gynecology, Rush University Medical Center, Chicago, Illinois, United States of America; 3 Department of Pathology, Rush University Medical Center, Chicago, Illinois, United States of America; 4 Department of Cell and Molecular Medicine, Rush University Medical Center, Chicago, Illinois, United States of America; 5 Department of Animal Science, University of Illinois Urbana-Champaign, Champaign, Illinois, United States of America; University of Alabama at Birmingham, UNITED STATES

## Abstract

Chronic inflammation fundamentally influences cancer risk and development. A mechanism of chronic inflammation is the formation of inflammasome complexes which results in the sustained secretion of the pro-inflammatory cytokines IL1β and IL18. Inflammasome expression and actions vary among cancers. There is no information on inflammasome expression in ovarian cancer (OvCa). To determine if ovarian tumors express inflammasome components, mRNA and protein expression of NLRP3 (nucleotide-binding domain, leucine-rich repeat family, pyrin domain containing 3), caspase-1, IL1β, and IL18 expression in hen and human OvCa was assessed. Chicken (hen) OvCa a valid model of spontaneous human OvCa. Hens were selected into study groups with or without tumors using ultrasonography; tumors were confirmed by histology, increased cellular proliferation, and expression of immune cell marker mRNA. mRNA expression was higher for hallmarks of inflammasome activity (caspase-1, 5.9x increase, p = 0.04; IL1β, 4x increase, p = 0.04; and IL18, 7.8x increase, p = 0.0003) in hen OvCa compared to normal ovary. NLRP3, caspase-8 and caspase-11 mRNA did not differ significantly between tumor and non-tumor containing ovaries. Similar results occurred for human OvCa. Protein expression by immunohistochemistry paralleled mRNA expression and was qualitatively higher in tumors. Increased protein expression of caspase-1, IL1β, and IL18 occurred in surface epithelium, tumor cells, and immune cells. The aryl hydrocarbon receptor (AHR), a potential tumor suppressor and NLRP3 regulator, was higher in hen (2.4x increase, p = 0.002) and human tumors (1.8x increase, p = 0.038), suggesting a role in OvCa. Collectively, the results indicate that inflammasome expression is associated with hen and human OvCa, although the NLR sensor type remains to be determined.

## Introduction

Chronic inflammation is associated with cancer risk and is an element of tumor development [1–4]. There is increasing evidence that inflammasome formation promotes a chronic, pro-inflammatory environment [5, 6]. However, the role of inflammasomes in cancer progression remains unclear since inflammasome expression varies among tumor types and pro- and anti-tumor effects occur in different cancers [6, 7].

Inflammasomes are large multi-protein complexes, composed of a sensor (receptor), an effector and an adaptor protein that control the activation of caspase-1 [8]. Activated caspase-1 stimulates the production of IL1β and IL18. Inflammasomes are categorized based on their sensor types and include NLRP1, NLRP3, NLRC4, AIM2, and NLRP6 [6], each activated by different signals [9]. The NLRP3 inflammasome is the best-characterized inflammasome [10]. It is primarily cytoplasmic and contains the sensor NLR (nucleotide-binding oligomerization domain [NOD]-like receptors), the adaptor protein ASC (apoptosis-associated speck-like protein containing a caspase activation and recruitment domain) and the effector protein caspase-1. The NLRP3 inflammasome has a broad range of activators such as dsRNA, extracellular ATP or asbestos [11]. NLRP3 inflammasome assembly activates caspase-1 which then converts pro-interleukin-1β (IL1β) and pro-interleukin-18 (IL18) to active IL1β and IL18 [5, 8]. IL1β and IL18 are apex regulators of pro-inflammatory pathways. A consequence of inflammasome activation is pyroptosis, a form of programmed lytic cell death that is distinct from apoptosis [12].

The NLRP3 inflammasome is involved in tumor development, although the precise role of the NLRP3 inflammasome is unclear [9, 13] since the cytokines it produces suppress some cancers, while they facilitate tumorigenesis of other cancers. For example, in hepatocellular carcinoma, patients with *low* expression levels of NLRP3 inflammasome components had a worse prognosis [14]. Colitis-associated cancer was higher in NLRP3 knockout mice models; the increased tumor burden was correlated with attenuated levels of tumor IL-1β and IL-18 [15]. In contrast, *increased* NLRP3 inflammasome activity promotes skin and breast cancer [7]. There is no information on inflammasome expression in ovarian tumors.

The molecular regulation of the NLRP3 inflammasome involves both positive and negative regulatory pathways, and regulation occurs at the transcriptional and post-translational levels [10, 16]. The aryl hydrocarbon receptor (AHR) negatively regulates NLRP3-mediated caspase-1 activation and IL-1β secretion in macrophages by inhibiting NLRP3 transcription [17]. AHR expression is increased in multiple cancers [18]. It is expressed in human ovarian cancer, and the endogenous AHR ligand, 2-(1'H-indole-3'-carbonyl)-thiazole-4-carboxylic acid methyl ester (ITE), inhibits ovarian cancer cell proliferation and migration *in vitro* [19]. In turn the tumor suppressor AHRR (aryl hydrocarbon receptor repressor) inhibits AHR and reduces inflammation and cancer progression [18, 20]. In normal cells, AHR activation induces AHRR which then negatively regulates AHR. In cancer cells, the AHRR-AHR feedback loop is interrupted by methylation of the AHRR promoter which blocks its expression. The absence of AHRR results in the induction of genes associated with tumorigenesis and cancer progression [20].

The study objective was to determine if the NLRP3 inflammasome components (NLRP3, caspase-1), and the corresponding cytokine products, IL1β and IL18, were increased in ovarian tumors. AHR and AHRR were assessed to determine if the expression correlated with the inflammasome in ovarian cancer. We used the hen, an animal model of spontaneous human ovarian cancer, that has been shown to have validity as a model of ovarian cancer based on histological, biochemical and immunologic similarities to human ovarian cancer [21–25]. The Human Protein Atlas suggests human ovarian tumors express NLRP3, caspase-1, IL1β and IL18 [26, 27]. But there is limited experimental information on expression of NLRP3 inflammasomes in human ovarian tumors. Therefore human samples were examined for comparison to observations in hen ovaries to provide an indication of the generalizability of hen results to human OvCa.

## Materials and methods

**Tissues:** White Leghorn hens (3 years old, strain W96) were housed at the poultry farm of the University of Illinois at Urbana-Champaign, Department of Animal Science. Food and water were provided *ad libitum*, and hens were maintained on a 17:7 hours (light: dark) schedule. Ovarian morphology was evaluated using transvaginal ultrasound as described previously (26); the data were used to select hens with normal ovaries (n = 8) or ovarian tumors (n = 10). Transvaginal ultrasound is not associated with pain or suffering and does not require anesthesia or analgesia. Hens were rapidly euthanized by standard cervical dislocation and the ovaries were removed and immediately placed in ice-cold phosphate buffered saline (PBS; pH 7.4). This study complied with the recommendations of the Guide for the Care and Use of Laboratory Animals of the National Institutes of Health [28]. The Institutional Animal Care and Use Committees at Rush University Medical Center (number 08–011) and the University of Illinois (number 05147) approved the study protocol.

Human tissue for mRNA from patients with normal (n = 4) ovaries or ovarian tumors (n = 8) was obtained with written informed consent following a protocol approved by the Internal Review Board at Rush University Medical Center during December 2004 through February 2009. The inclusion criteria were primary epithelial ovarian cancer from previously untreated patients undergoing debulking surgery. The average age of the cancer patients was 60±9 years. The sample is representative of the Chicago area population. The demographics of the cancer patients is shown in **S1 Table**. For mRNA controls, normal ovarian tissue was obtained from patients (ages 30–70) following complete hysterectomy from the National Disease Research Interchange (NDRI) without patient demographic data.

For immunohistochemistry, human tissue arrays containing cancer and control tissue were obtained commercially (catalogue numbers T111, T112a, T112b, MC241, OV242, T114, T113a, OV802at; US BioMax, Derwood, MD). Normal tissue sections were from a combination of cancer adjacent areas (1.5 cm away from tumor), and normal non-cancer containing ovaries (unmatched). Patient characteristics were provided by US BioMax (**S1 Table)**. US BioMax obtained written informed consent. Information on the population of origin represented by the commercial arrays was not available from US Biomax, and thus, its application to the general population is not known. For this study, the inclusion criteria were primary epithelial ovarian cancer from previously untreated patients undergoing debulking surgery; exclusion criteria included germ cell or other stromal tumors.

### Protein analysis by histology and immunohistochemistry

Ovaries were fixed in PBS-buffered formalin (10%) and embedded in paraffin. Hematoxylin and Eosin (H&E) stained sections of ovary were analyzed for tumor type and stage based on similarities to human morphology, as we established in a previous report (28). In summary, early stage (I and II) tumors are confined to the ovary (stage I) or ovary and oviduct (stage II). Later stage tumors (III and IV) have local abdominal and peritoneal metastases with ascites (stage III) or distant metastases with profuse ascites (stage IV).

Protein expression was assessed by immunohistochemistry using the ABC method (Vectastain) in sections of human and hen ovarian and tumor tissue. Primary antibodies to human or chicken antigens (**S2 Table**) were used according to the manufacturer’s recommendations after establishing optimal dilutions. Characterization of normal and tumor ovary structures was based on our prior studies of ovarian tumor histology [23] and immune cells [24] in the hen. Briefly, the nuclei of tumor cells tend to show signs of activation such as enlarged nuclei, coarse chromatin with aggregates, etc. Immune cells were identified as “putative” immune cells based on morphology and an irregular distribution within the stroma and tissue layers.

Sections were immunostained using a kit with diaminobenzidine (DAB) as a substrate (Vector Labs, Burlingame, CA). Sections were washed (15 minutes), counterstained with Hematoxylin (Fisher Scientific, Rockford, IL), dehydrated, applied to slides, and dried (37°C, 16 hours). In preliminary procedures, sections of spleen and liver were assessed as positive controls to evaluate antibodies. Microscopy was performed on a Zeiss Axiovert or an AmScope B120 light microscope. Images were captured digitally. A pathologist reviewed the images.

### mRNA analysis

Human and chicken mRNA were measured by qRT-PCR as described previously (29–32). RNA was extracted with Trizol reagent (Invitrogen, Carlsbad, CA). The 260/280 absorbance ratio was used to assess RNA concentration and quality. Total RNA was treated with DNAse to remove genomic contamination, and 1.0 μg was used for first strand synthesis using a High Capacity cDNA Reverse transcription kit (Applied Biosystems Inc, Foster City, CA. Primers (**S3 Table**) were based on genes from the NCBI database; chicken primers were orthologues of human genes. Oligoperfect Designer software (Invitrogen; Carlsbad, CA) was used to design each primer and the endogenous actin control. 25 ng of the first strand was used for each PCR reaction as template. PCR comprised initial denaturation at 94°C for 3 minutes, followed by 35 cycles (each cycle at 94°C for 30 seconds, 57°C for 30 seconds and 72°C for 1 minute). PCR amplicons were separated in a 3% agarose gel and stained with ethidium bromide. The PCR product was purified (QIAquick PCR purification kit; Qiagen, Valencia, CA) and then sequenced (DNA sequencing facility, University of Illinois at Chicago; ABI BigDye Terminator in an ABI 3100 Genetic analyzer, Applied Biosystems Inc, Foster City, CA) using the same primers. The contour quantities of bands in gels (density of the band multiplied by the area of the band) were measured using Quantity One software (BioRad, Hercules, CA). The contour quantities of actin bands were used to normalize the contour quantities of other bands.

Quantitative Reverse Transcriptase-PCR (qRT-PCR) was carried out using SYBR green master mix in an ABI 7500 RT-PCR system and analyzed using the ΔCt method with chicken Actin as an internal control (Applied Biosystems). The ΔΔCt was determined by subtracting ΔCt of each sample from the average ΔCt of a normal ovary standard. The differences in mRNA expression levels were calculated as the fold change using the formula 2-ΔΔCt [29]. mRNA expression is the mean fold change (mFC) relative to normal tissue.

### Statistical analyses

Statistical tests were done using GraphPad Prism or SPSS (Chicago, IL). The Mann Whitney U test was used to determine if mean differences were significant with p<0.05 considered significant. Spearman correlation analysis was used to assess relationships between mRNA levels for MRC1LB vs. NLRP3 and for caspase-1 vs. IL1β.

## Results

### Characterization of normal and tumor-containing ovaries

**Fig 1:**
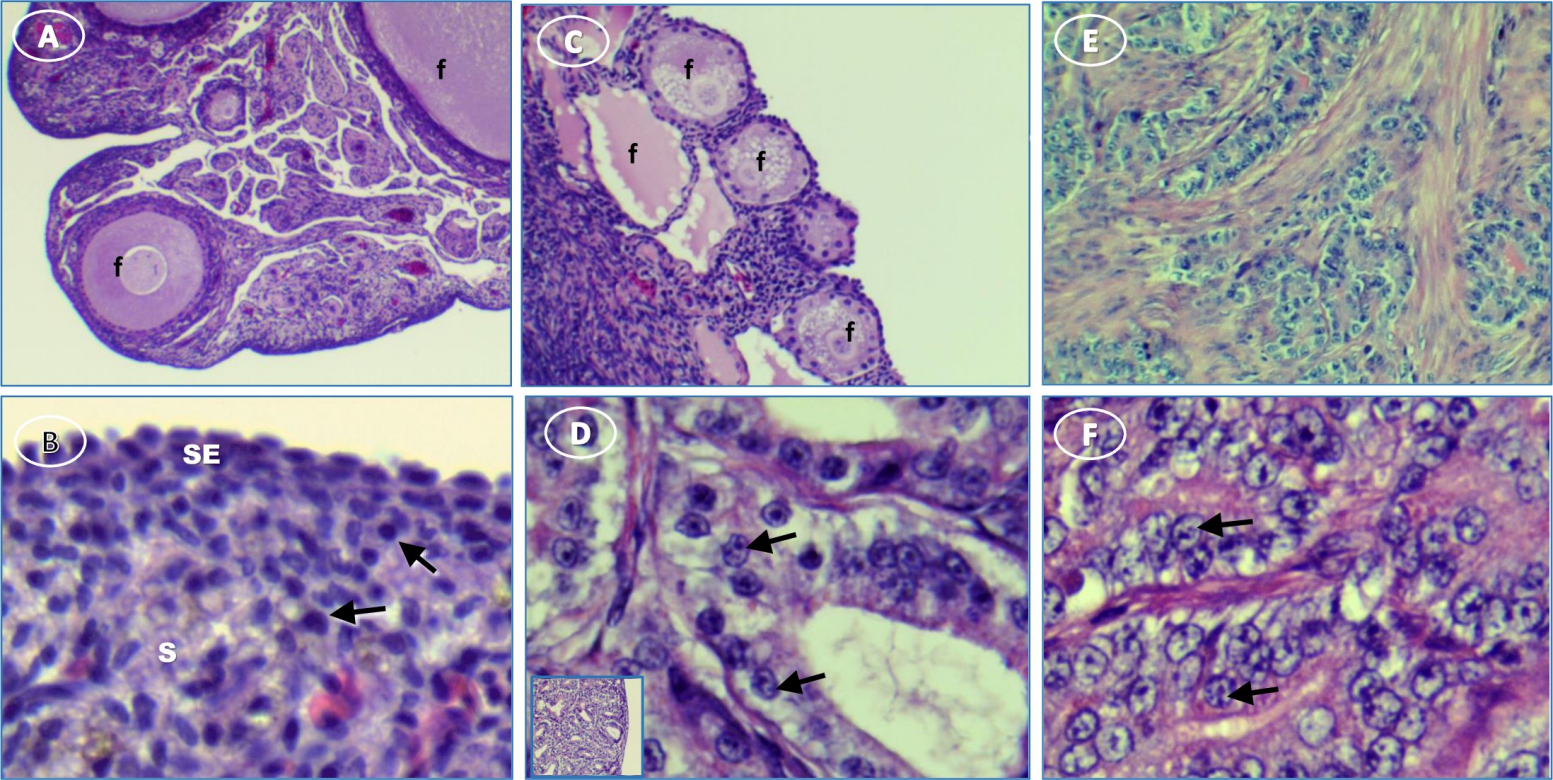
Hen ovary with and without tumors stained with H&E. (**A**) normal ovary with developing follicles (f); (**B**) normal ovary showing surface epithelium (SE) and cells with resting nuclei (arrows) in stroma (s); (**C**) ovary with ovarian tumor has degenerating follicles (f); (**D**) activated nuclei (arrows) in mucinous tumor showing [inset, low magnification showing overall morphology]; (**E**) serous tumor; (**F**) higher magnification of E showing activated nuclei (arrows). Original magnification: (A) 4X; (B) 4X [inset, 40X]; (C) 10X; (D) 40X [inset, 4X]; (E) 10X; (F) 40XTumors commonly have a proliferative cell profile. Consistent with the ultrasound designation, several indicators of proliferation, PCNA (Proliferating Cell Nuclear Antigen) (p = 0.04), WT1 (Wilms Tumor 1 protein) (p = 0.012) and EpCAM mRNA (3.7x; p = 0.0001) were significantly higher in ovarian tumors compared to normal ovaries in the hen.

Examples of the histology of ovaries determined to be normal or to contain a tumor by ultrasonography are shown in **Fig 1**. Normal ovaries contain developing follicles ranging from small primary to large pre-ovulatory follicles. Ovaries with tumors have few or no follicles and numerous degenerating follicles. All the ovaries classified as tumor-containing by ultrasound had either no follicles or variable follicle numbers. Follicles found in tumor-containing ovaries were typically not well developed or showed signs of degeneration.

**Fig 2:**
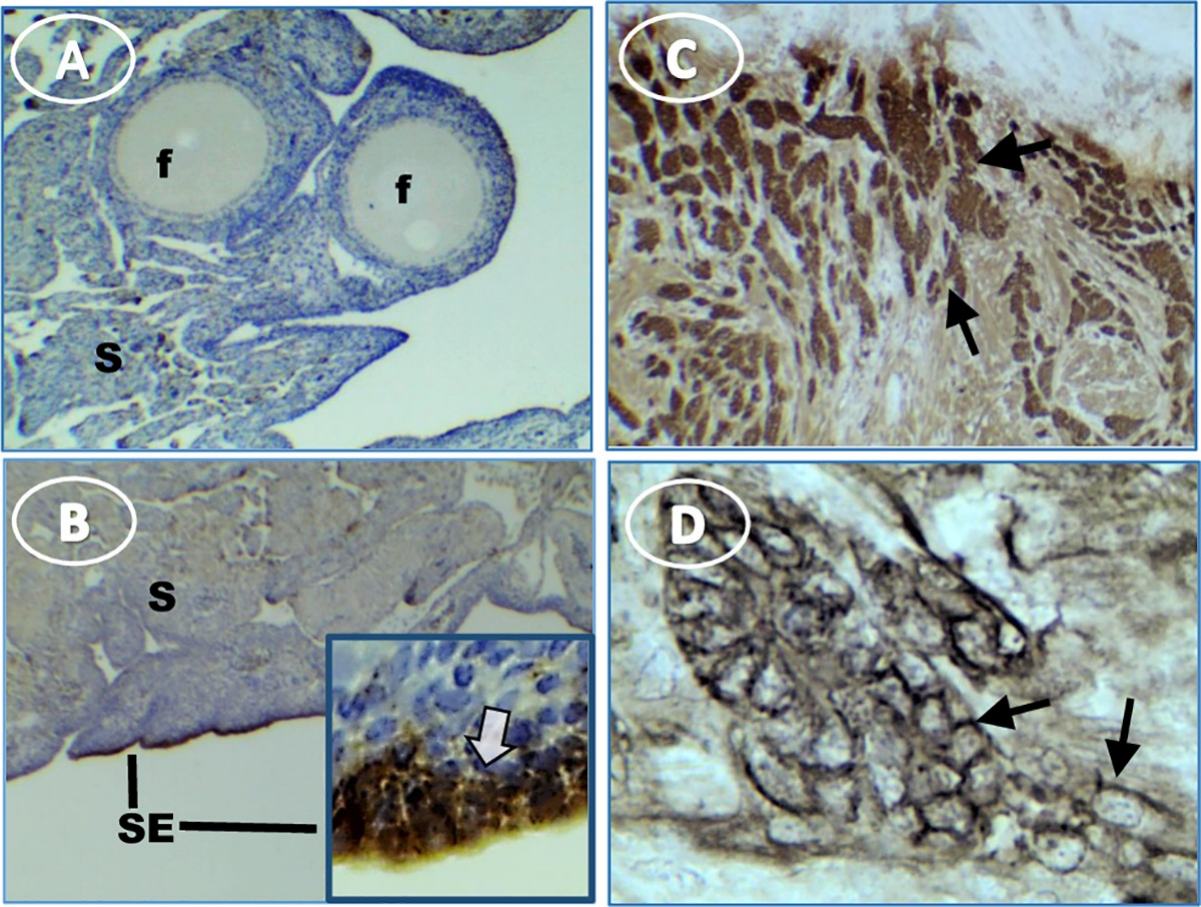
EpCAM expression in hen ovaries with and without tumors. (**A**) normal ovary expresses relatively little EpCAM, (**B**) EpCAM is highly expressed in normal ovary surface epithelium (SE); inset shows intense stain [white arrow] at higher magnification, (**C**) tumor cells express EpCAM (arrows), (**D**) higher magnification of ovarian tumor shows EpCAM is expressed primarily on membranes (arrows). Original magnification: (A) 4X; (B) 4X [inset, 40X]; (C) 4X; (D) 40X.

Tumor-containing ovaries stained intensely and extensively for EpCAM by immunohistochemistry (**Fig 2)**. EpCAM stain in normal ovaries was limited to surface epithelial cells (**Fig 2A & 2B**). In contrast, stain occurred on surfaces of tumor cells arranged in cords within the tumor stroma (**Fig 2C & 2D**).

Increased immune cell content is a hallmark of tumors. T cell mRNA expression was higher overall (CD3d, p = 0.003; CD45, p = 0.006; CD4, p = 0.0006), and there was a dramatic increase in chB6 (B cell) mRNA (median increase 23x; p = <<0.0001) in hen tumors compared to normal ovary as we described previously [24]. Among T cell subsets, the expression of CD8a (p = 0.055) approached significance but CD8b (p>0.5) did not differ significantly from expression in the normal ovary. MRC1L-B, a homologue of the mammalian mannose receptor identifies chicken macrophages [30]; MRC1L-B mRNA was 2-fold higher in tumor-containing ovaries (p = 0.002).

### Inflammasome mRNA expression

**Fig 3:**
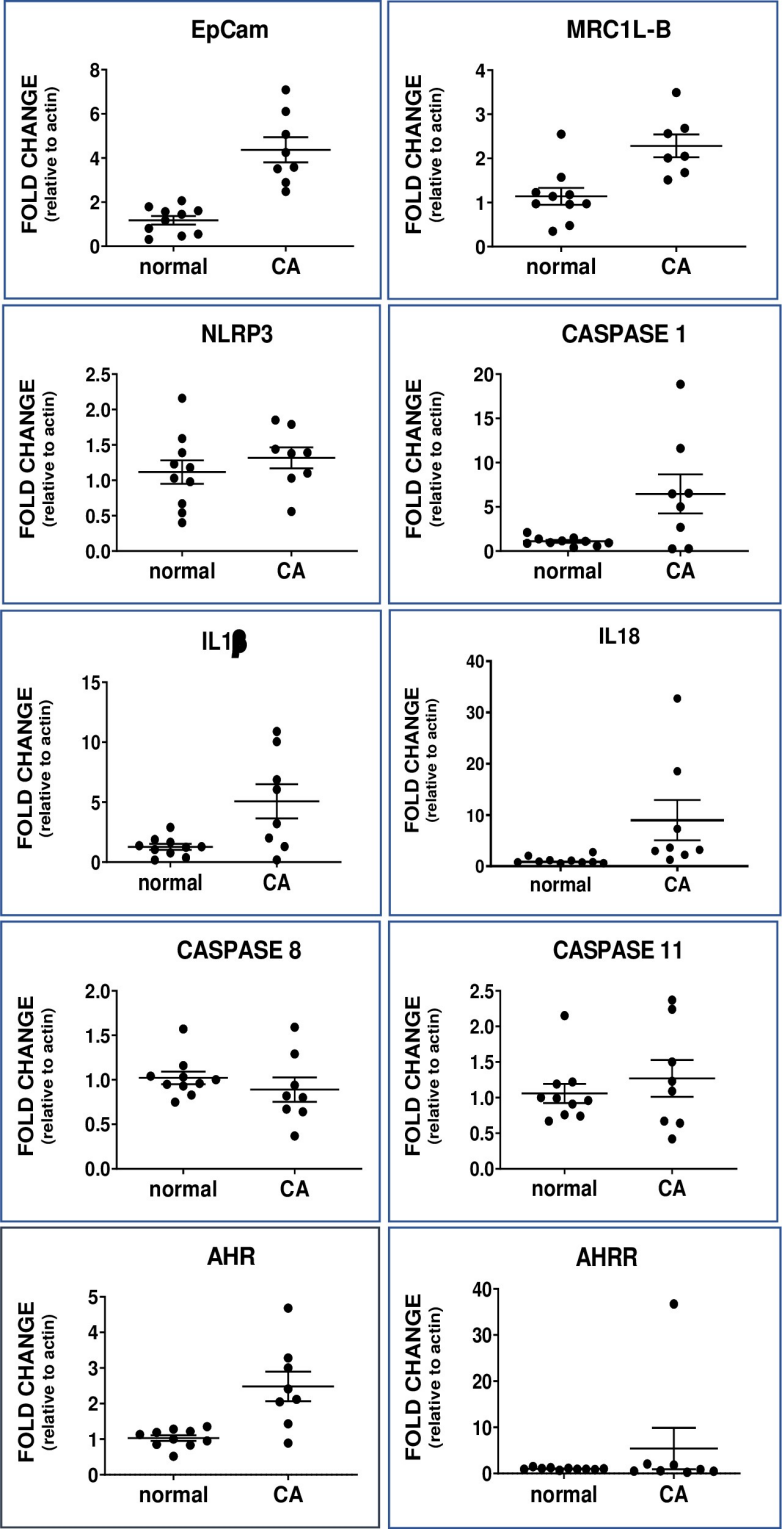
Dot plot of mRNA levels in hen normal ovary (normal) and ovarian cancer (CA). EpCAM, a proliferation marker, differed significantly (p<0.001). MRC1L-B, a marker for macrophages, differed significantly (p = 0.002). The difference between mRNA of inflammasome components differed significantly for caspase-1 (CASP1) (p = 0.04), IL1β (p = 0.04) and IL18 (p = 0.003) but not NLRP3 (p = 0.3) between normal and cancer ovaries; other caspase mRNAs caspase-8 (CASP8) (p = 0.17) and caspase-11 (CASP11) (p = 0.65) did not differ. AHR (p = 0.002) but not AHRR (p = 0.5) mRNA expression differed between normal and cancer ovaries. Note: Y-axis range differs among graphs; values are mean fold change relative to actin ± SEM.

The changes for the inflammasome mRNAs were parallel in chicken and human and ovaries and tumors. In hen ovaries (**Fig 3**), caspase-1 mRNA was 5.9x higher (p = 0.04) in tumors than in normal ovaries. Furthermore, caspase-1 was correlated with IL1β (r = 0.74, p = 0.046) in tumor-containing hen ovaries. Caspase-8 was assessed for comparison since it has a central role in apoptosis (35, 36); it did not differ (p = 0.2) between normal and tumor containing ovaries. Caspase-11 drives an alternate NLRP3 pathway (37) and did not vary (p = 0.6). Although the mean difference was 18% higher in ovarian tumors, changes in NLRP3 mRNA did not reach statistical significance (p = 0.3). The mRNA expression of the inflammasome products IL1β (4x; p = 0.02) and IL18 (7.8x; p = 0.0003) was increased significantly in hen tumors compared to normal ovary. Inflammasome responses usually are associated with macrophages. Although NLRP3 expression did not increase significantly, as expected it was highly positively correlated with the macrophage marker MRC1LB (r = 0.90, p = 0.005).

**Fig 4:**
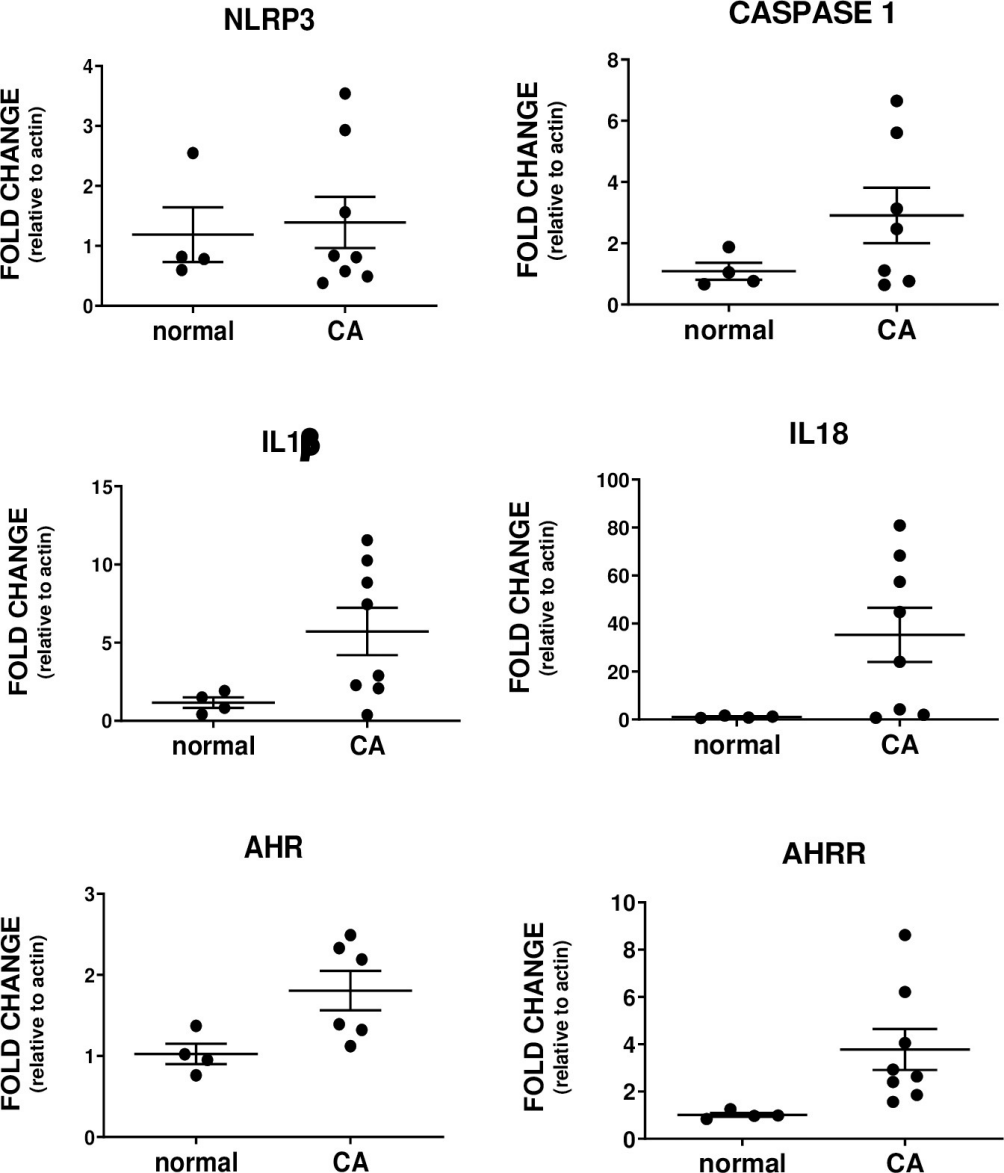
Dot plot of mRNA levels in human normal ovary (normal) and ovarian cancer (CA). The differences for mRNA of inflammasome components were significant for caspase-1 (CASP1) (p = 0.05), IL1β (p = 0.04) and IL18 (p = 0.04) but not NLRP3 (p = 0.06). AHR (p = 0.038) and AHRR (p = 0.04) mRNA levels differed significantly. Note: Y-axis range differs among graphs; values are mean fold change relative to actin ± SEM.

Similar to the chicken, CASP1 (2.7x; p = 0.05), IL1β (4.9x; p = 0.04) and IL18 (33x; p = 0.02) mRNA were significantly higher in human ovarian tumors (**Fig 4**). NLRP3 increased 1.2x (p = 0.06) but the difference from normal ovary did not reach significance.

### AHR and AHRR mRNA expression

AHR (2.4x, p = 0.002) and AHRR (2.7x, p = 0.4) expression were higher in hen ovaries with tumors compared to normal ovaries (**Fig 3**). Similarly, AHR (1.8x, p = 0.04) and AHRR (3.7x, p = 0.004) expression were higher in human tumors compared to normal ovaries (**Fig 3**).

**Fig 5:**
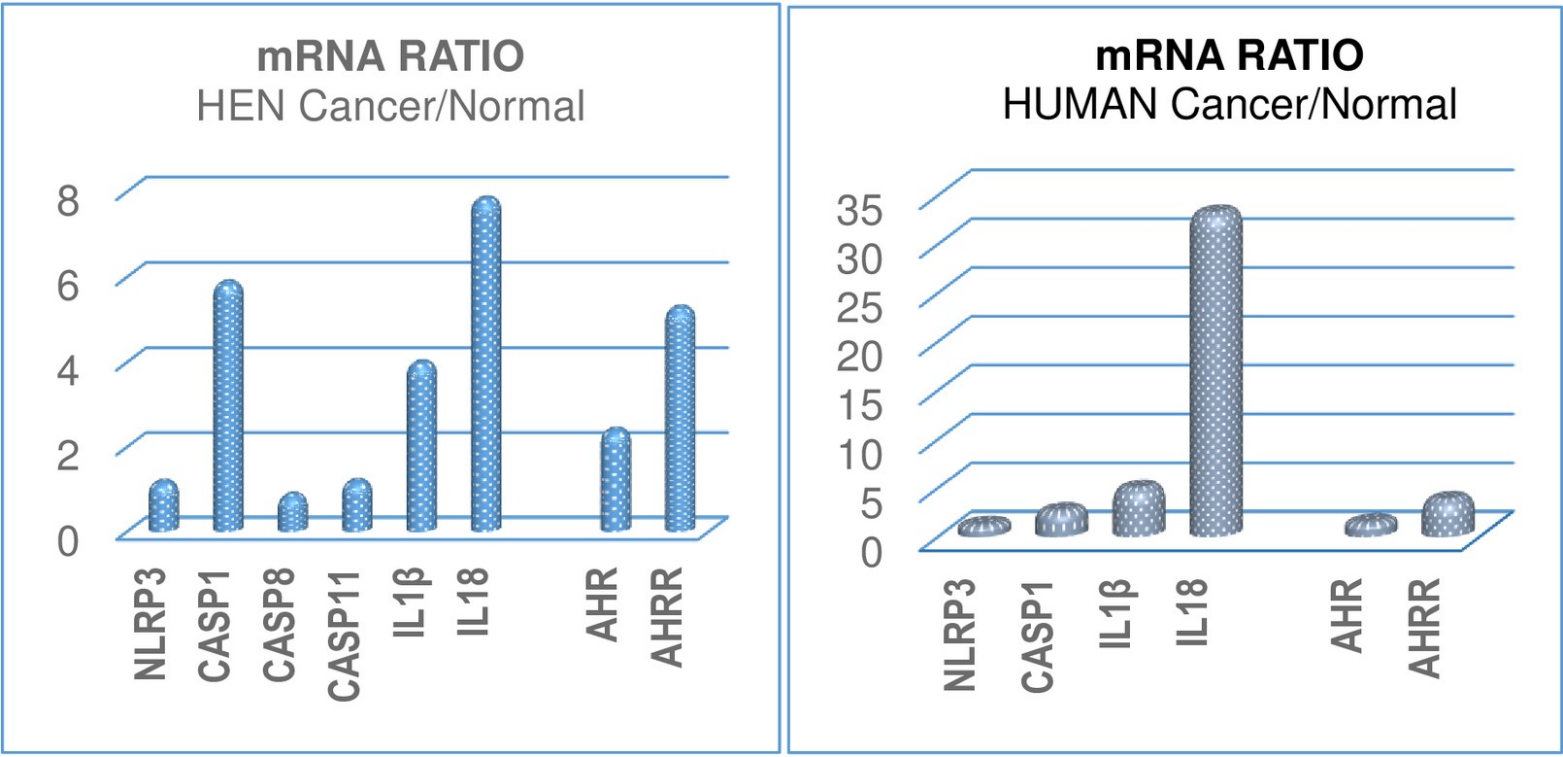
**Summary of changes in mRNA expression** shown as the **ratio of cancer/normal** values for NLRP3, caspase-1, IL1β, IL18, AHR and AHRR in (**left**) **hen** and (**right**) **human** ovaries to show the relative changes. Note: Y-axis range differs between graphs.

The changes in mRNA expression in ovarian cancer relative to control are summarized for the chicken and human in **Fig 5**.

### Inflammasome protein expression

**Fig 6:**
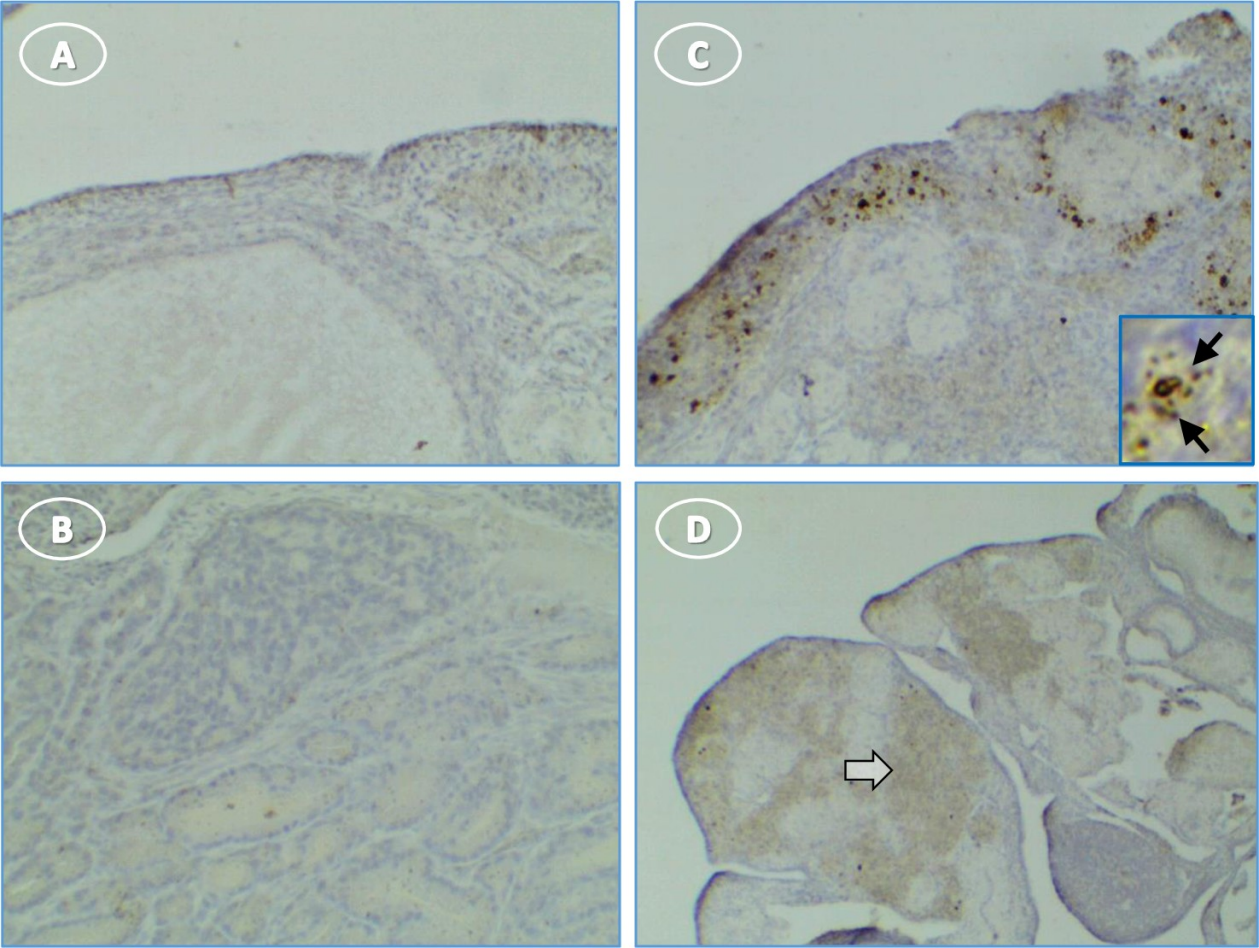
NLRP3 expression in hen ovary with and without tumors is low. (**A**) normal ovary without NLRP3 expression; (**B**) control tumor section without primary antibody is not stained; (**C**) immune cells in a tumor ovary stain for NLRP3; inset shows punctate cellular stain (arrow); (**D**) tumor cells are occasionally lightly stained (white arrow). Original magnification: (A) 10X; (B) 10X; (C) 10X [inset, 40X]; (D) 4x.

NLRP3 staining was not evident in normal ovaries (**Fig 6A**). There was light and diffuse cytoplasmic staining for NLRP3 in tumor stromal cells, with little or no staining of surface epithelial cells (**Fig 6C and 6D**). The cytoplasm of small groups of individual cells that resemble infiltrating immune cells stained for NLRP3 (**Fig 6C**). These cells had variable levels of diffuse and dark punctate cytoplasmic stain.

**Fig 7:**
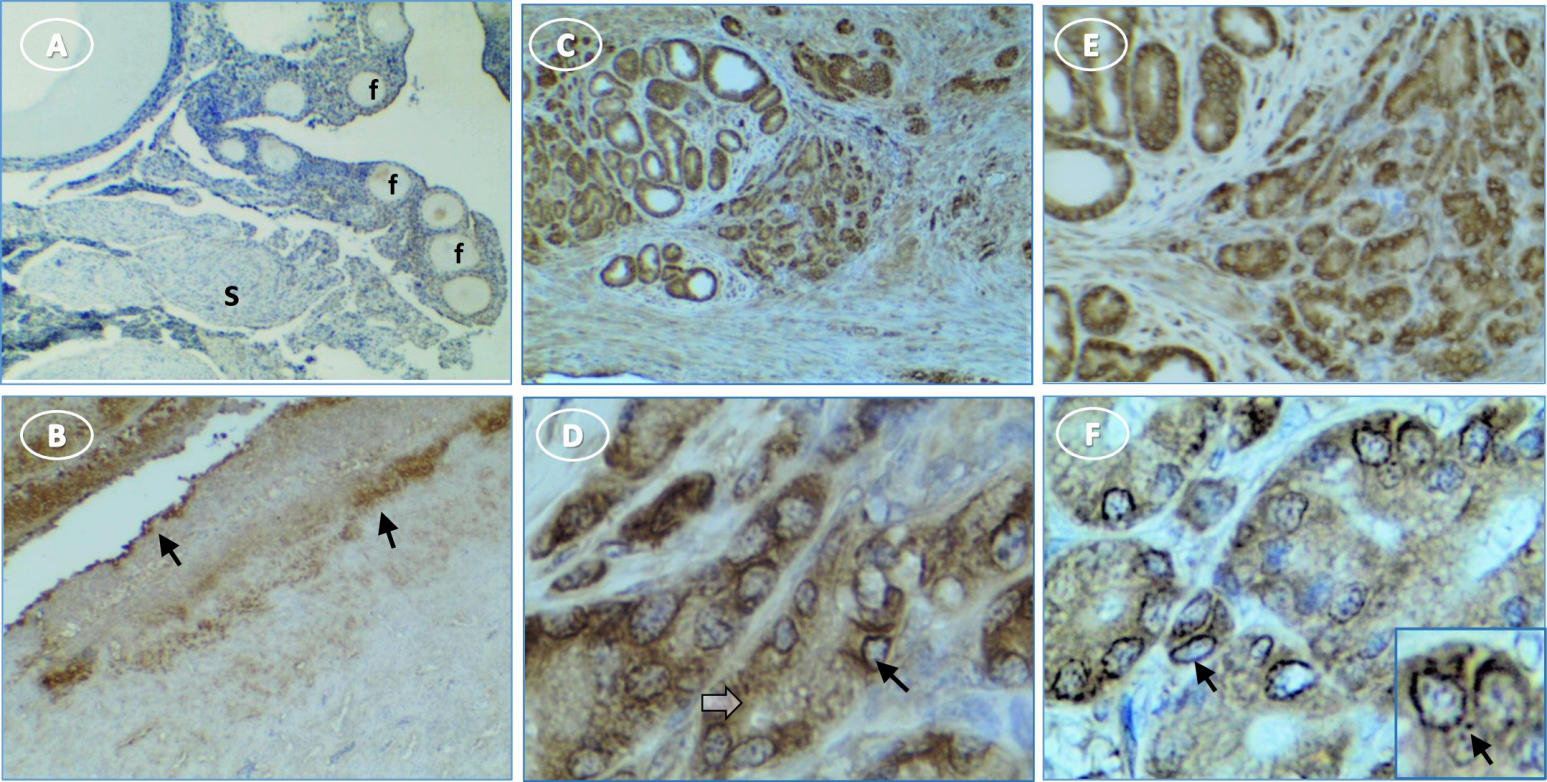
More caspase-1 is expressed in hen ovary with tumors than in normal ovary. (**A**) normal ovary without stain in follicles (f) or stroma (s); (**B**) normal ovary shows surface epithelium (arrow) staining; (**C, E**) intense stain in cords of tumor cells; (**D, F**) higher magnification shows perinuclear (arrow) and diffuse cytoplasmic stain (white arrow) [inset, punctate perinuclear stain (arrow)]. Original magnification: (A) 4X; (B) 4X; (C) 4X; (D) 40X; (E) 4X; (F) 40X [inset, 60X].

Caspase-1 expression was limited in normal ovaries to the surface epithelium and the layer immediately under the surface epithelium (**Fig 7A and 7B**). In tumor ovaries, caspase-1 staining was intense throughout the section. Staining in the cytoplasm of presumed immune cells varied from diffuse to punctate bodies (**Fig 7C–7F**). In tumor cells, caspase-1 staining occurred in the cytoplasm, occasional peri-nuclear sites and some punctate cytoplasmic structures (**Fig 7E and 7F**).

**Fig 8:**
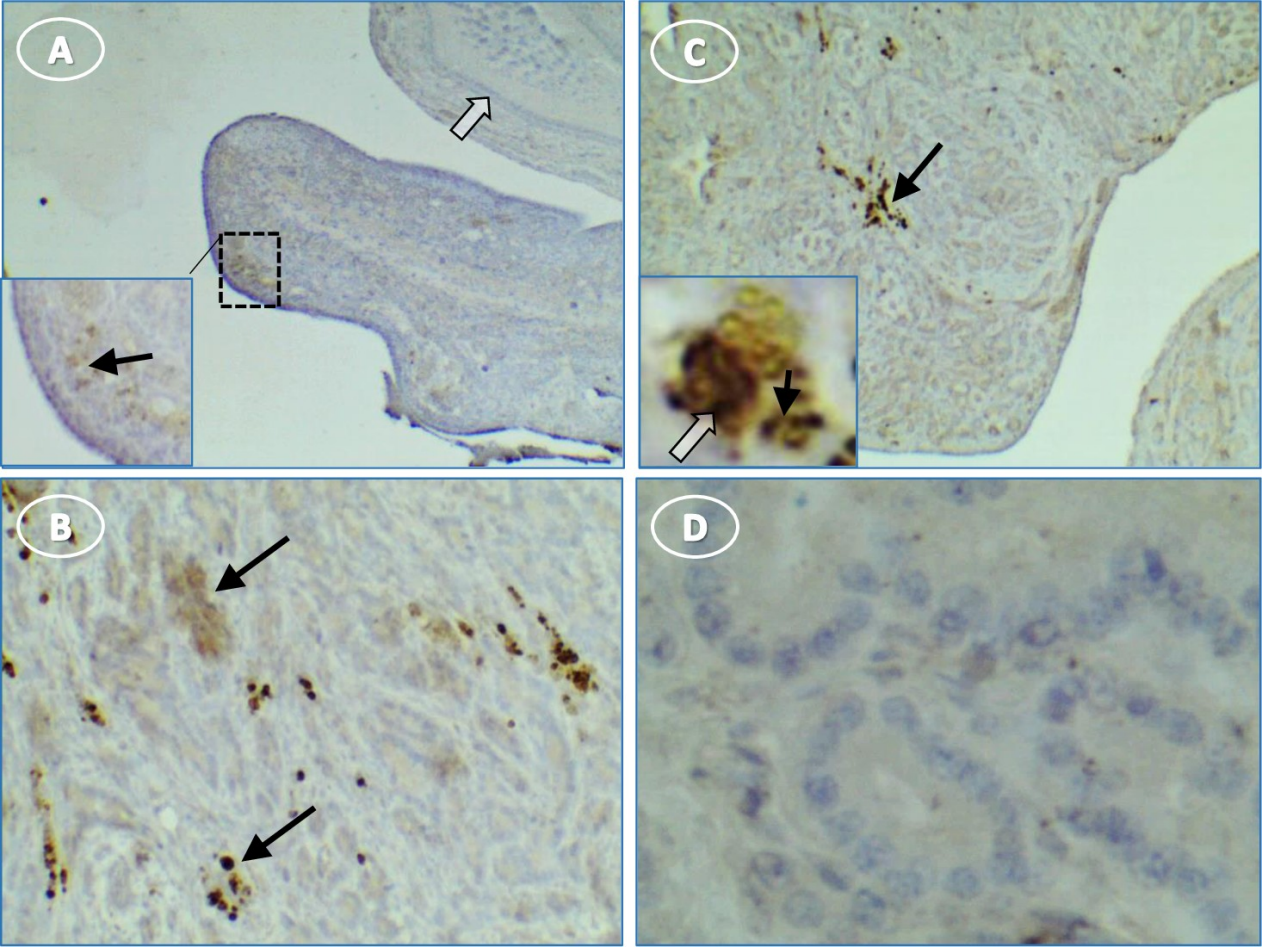
Hen ovary with and without tumors stained for IL1β. (**A**) normal ovary without stain except some faint groups of cells [inset, higher magnification of A showing faint cells (arrow) near surface epithelium]; (**B**) stained groups (arrows) of immune cells in tumor ovary; (**C**) immune cell groups in tumor stroma and higher magnification inset showing punctate stain in cell cytoplasm (black arrow) and nuclear stain (white arrow); (**D**) control section of tumor without primary antibody is not stained. Original magnification: (A) 4X [inset, 10X]; (B) 10X; (C) 4X [inset, 40X]; (D) 40X.

Little IL1β staining of the normal ovary was seen although there were patches of faint stain adjacent to the surface epithelium (**Fig 8A**). IL1β was expressed predominantly in clusters of cells that resembled migrating immune cells throughout the stroma of tumor ovaries (**Fig 8B and 8C**). There were also random areas of light stromal cell/tumor cytoplasmic staining (**Fig 8B**). No IL1β staining occurred in control sections when the primary antibody was omitted (**Fig 8D**).

**Fig 9:**
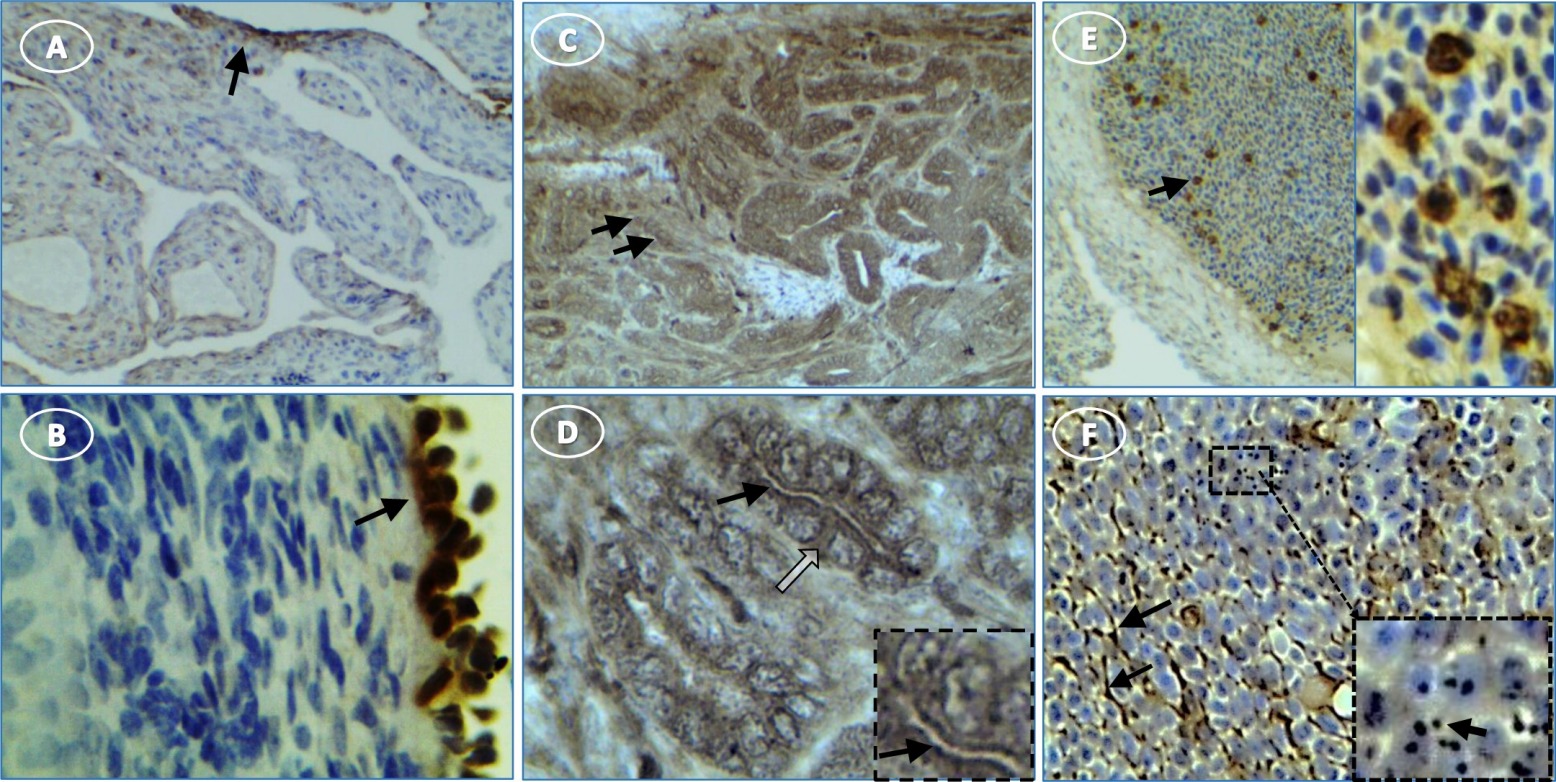
IL18 expression in hen ovary with and without tumors. (**A**) normal ovary with no stain in stroma but abundant surface epithelium stain (arrow); (**B**) higher magnification of A showing cytoplasmic cell staining in surface epithelium of normal ovary (arrow); (**C**) IL18 is expressed in cords of tumor cells (arrows); (**D**) higher magnification shows staining is primarily cytoplasmic (white arrow) and along cell surfaces (black arrow and inset); (**E**) (left panel) blood vessel with stained immune cells (arrow) and unstained nucleated red blood cells and (right panel) higher magnification of same area; (**F**) some tumor areas have extracellular (presumably secreted) IL18 which appears as aggregates and single points in extracellular spaces (black arrows) as shown at higher magnification inset. Original magnification: (A) 10X; (B) 40X; (C) 10X; (D) 40X; (E) 4X, left, 40X, right; (F) 4X [inset, 40X].

IL18 rarely was expressed in the normal ovary except in the surface epithelium (**Fig 9A and 9B**). In tumors, IL18 staining was strong and occurred throughout the tissue, in tumor cell cytoplasm with various intensities in ovarian cells and immune cells (**Fig 9C–9F**). Also, some material that appears to be secreted was evident as extracellular strands and aggregates (**Fig 9F**).

**Fig 10:**
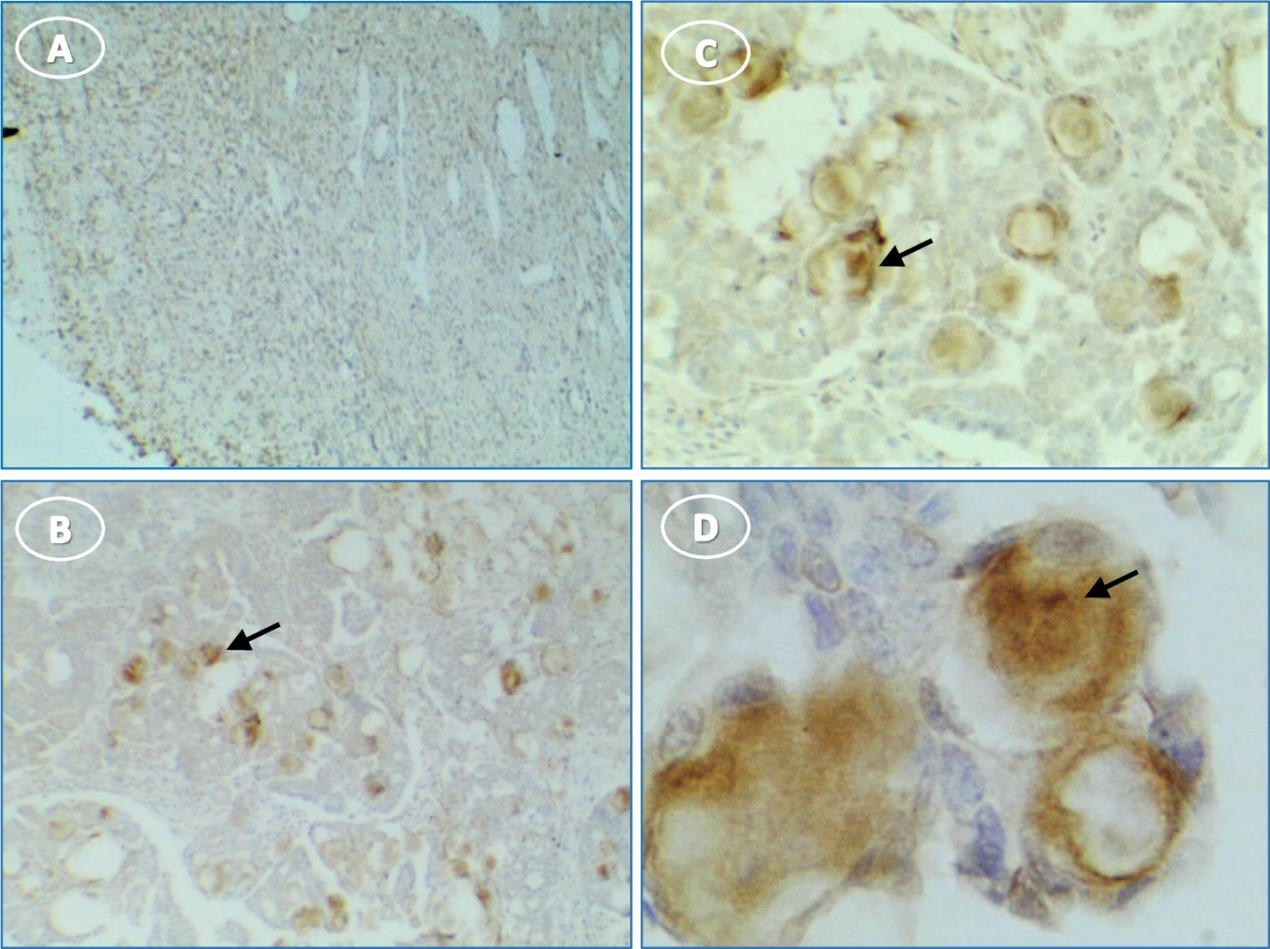
NLRP3 stain is minimal in human OvCa; (**A**) normal ovary; (**B** and **C**) occasional stained cells (arrow) associated with tumor; (**D**) higher magnification of (**C**) showing aggregated cytoplasmic stain. Original magnification: (A) 4X; (B) 4X; (C) 10X; (D) 40X.

**Fig 11:**
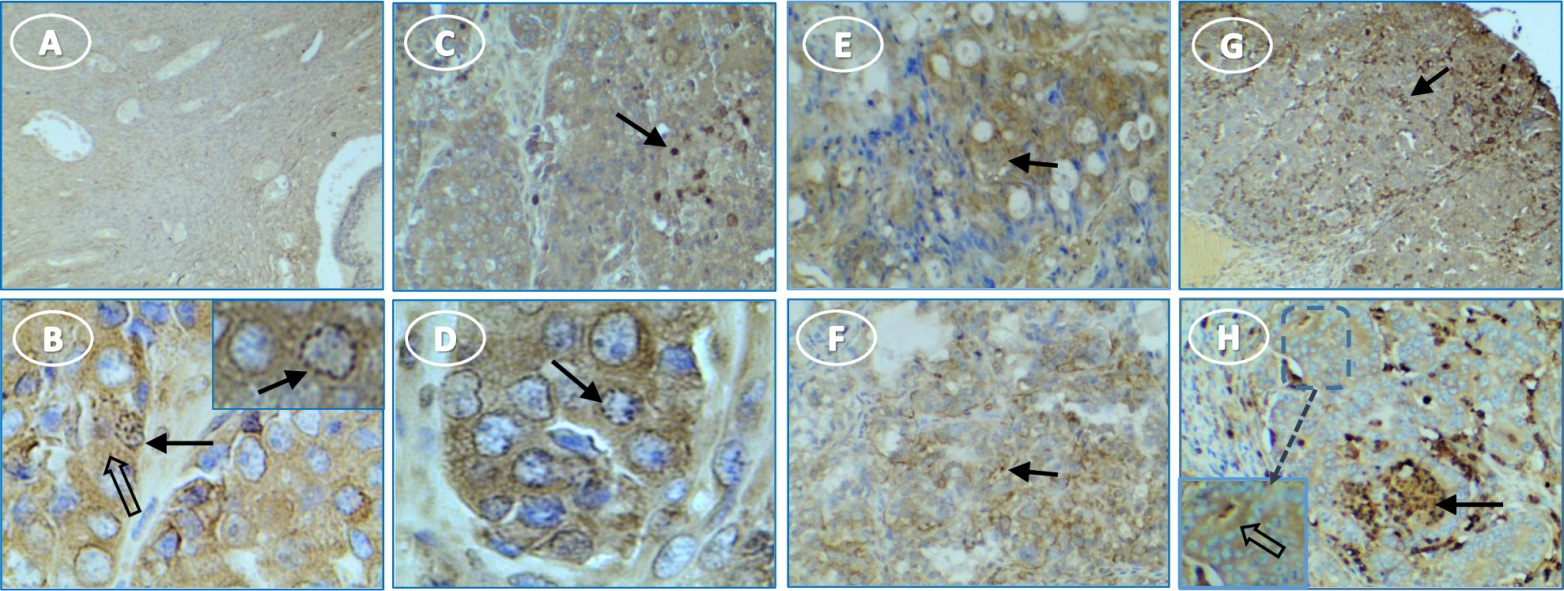
Caspase-1 expression occurs in tumor cells and immune cells in multiple human ovarian tumor types. (**A**) normal ovary without stain; (**B**) serous OvCa has punctate (arrow) and diffuse (white arrow) cytoplasmic stain in tumor cells; (**C**) serous OvCa showing dark stain in immune cells (arrows) among lighter stained tumor stroma; (**D**) serous OvCa showing punctate (arrow) and diffuse cytoplasmic (white arrow) stain in tumor cells; (**E**) diffuse cytoplasmic stain in mucinous OvCa; (**F**) light stain in clear cell tumor (arrows); (**G**) endometrioid tumor staining primarily in immune cells (arrows); (**H**) endometrioid tumor cell stain in cords (arrow) [inset, higher magnification shows tumor cell cytoplasmic stain (arrow)]. Original magnification: (A) 4X; (B) 10X [inset, 40X]; (C) 4X; (D) 40X; (E) 10X; (F) 4X; (G) 4X; (H) 4X [inset, 40X].

**Fig 12:**
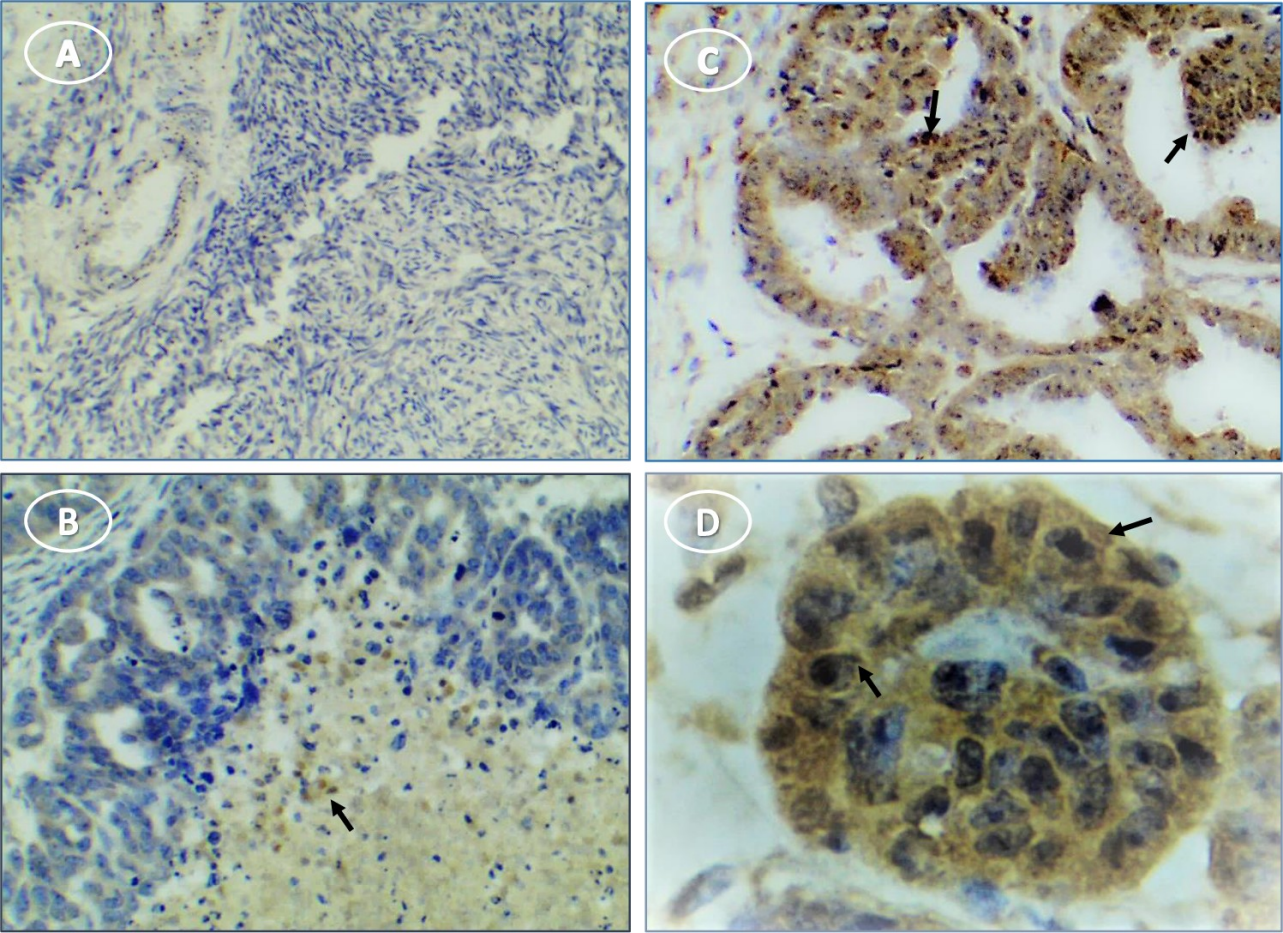
IL1β expression in human tumor and immune cells. (**A**) normal ovary is unstained; (**B**) very lightly stained cells (arrow) at a tumor edge; (**C**) stained tumor cells (arrow); (**D**) stained cells of C at higher magnification showing cytoplasmic (arrow) stain. Original magnification: (A) 10X; (B) 10X; (C) 10X; (D) 40X.

**Fig 13:**
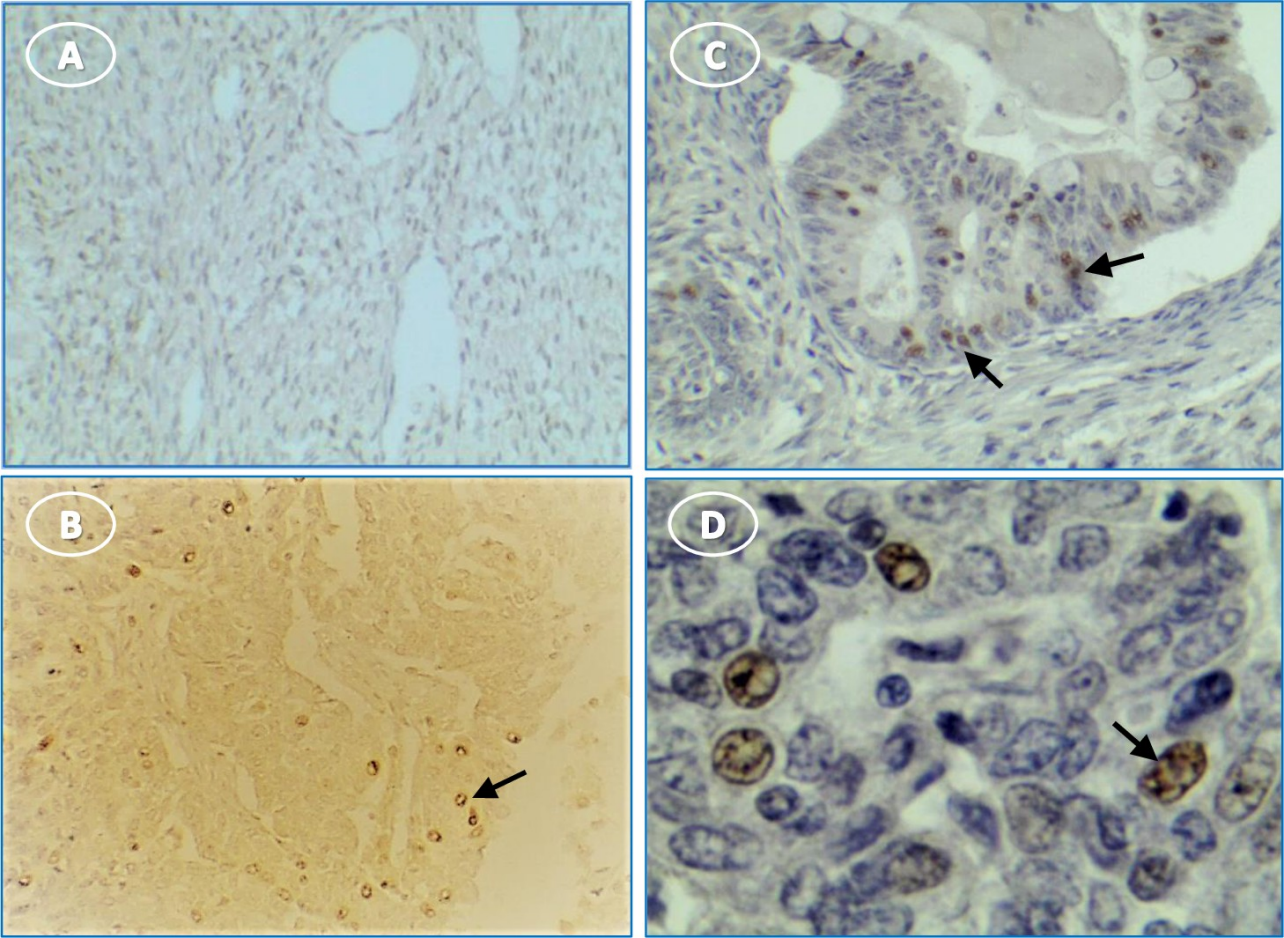
IL18 expression occurs predominantly in immune cells in human ovary with tumors. (**A**) unstained normal ovary; (**B**) stained cells in tumor section without hematoxylin stain; (**C**) stained immune cells among tumor cells; (**D**) enlarged selection from image **C** at higher magnification to show cytoplasmic stain of invading presumptive immune cells. Original magnification: (A) 4X; (B) 10X; (C) 10X; (D) 40X. Arrows indicate immune cells.

Inflammasome components also were expressed in human ovarian tumors. NLRP3 staining was negligible or faint in tumor stroma and stronger in scattered cells throughout the tissue (**Fig 10**). Caspase-1 staining was intense in most stages and types of tumor cells, with punctate stain around nuclei and on some cell surfaces (**Fig 11**). Moderate to light IL1β staining occurred fairly uniformly in tumor cell cytoplasm (**Fig 12**). IL18 stained cells were scattered through tumor stroma and adjacent to the tumor edge (**Fig 13**). Minimal expression of NLRP3, caspase-1, IL1β, or IL18 occurred in normal tissue (**Figs 10–13**).

## Discussion

The expression of caspase-1, IL1β, and IL18, hallmark indicators of inflammasome expression, was higher overall in the presence of tumors in hen and human ovaries compared to normal ovaries. This finding is important since inflammasomes are a druggable target. Drugs targeting the inflammasome could reduce inflammation and cancer progression [13]. In summary, the protein expression of the inflammasome components detected by immunohistochemistry occurred in both immune cells and tumor cells. Expression patterns of protein were similar in chicken and human ovarian tumors. AHR, a potential inflammasomes regulator, was similarly higher in hen and human ovarian cancer.

This is the first report of the general expression of inflammasomes in ovarian cancer. Chang [31] used expression profiling to identify mechanisms involved in the malignant transformation of endometriosis to ovarian cancer; 18 genes were identified, including NLRP3, IL-1B, and IL-18, which implicated inflammasomes. They found a significant correlation between the expression levels of the target genes and the progression-free patient survival; high expression levels of AIM2 and NLRP3 were significantly correlated with low progression-free survival.

The mRNA and protein expression results were correlated qualitatively for each of the measures. For example, caspase-1 mRNA expression was dramatically higher, and the protein expression detected by anti-caspase-1 antibody was intense in human and hen tissue. However, within each tissue section, the expression was not uniform; it varied by cell type and areas within the sections. In some cases, such as for IL18, immune cells stained intensely by immunohistochemistry while ovarian tumor cell cytoplasm stained moderately.

For caspase-1 and IL18, the pattern of stained cells was similar in hen and human tumor tissue. The results for IL1β were similar except that human tumor tissue stained more often than in the hen, which might be explained by differences in the antibody efficacy for hen and human immunohistochemistry. Also, samples were selected randomly from the hen ovary for different analyses. Since the tissue is not homogeneous, samples from ovaries with earlier stage tumors may not have similarly represented tumor cells, which could potentially contribute to variations in the immunohistochemistry staining and differences between hen and human tissue. Despite these considerations, the mRNA and protein results were remarkably similar, both within hens and between hen and human ovaries.

NLRP3 mRNA and protein levels were low and did not differ between normal and tumor ovaries. Although surprising in light of the elevated caspase-1, IL1β, and IL18, this is consistent with other reports that NLRP3 expression is relatively low in various cell types [16]. However, inflammasome reactions often occur in macrophages [32]. An influx of macrophages into tumors is well documented [33–36] and we showed increased mRNA for the chicken macrophage marker in tumor ovaries. Although NLRP3 expression did not differ between normal and tumor ovaries, NLRP3 levels were significantly correlated with the macrophage marker MRCL1B.

A possible explanation for the lack of increased NLRP3 expression, despite elevated caspase-1, IL1β and IL18, is that only specific locations have cells with activated NLRP3 (i.e., a cluster of invading immune cells at the tumor/tissue interface) and tissue was from regions without altered NLRP3. However, this is not likely since different samples were used for immunohistochemistry and mRNA. Furthermore, the result was the same in hen and human. Moreover, if the NLRP3 result were due to a technical issue, such as an ineffective antibody, or an inappropriate PCR primer, then the results of hen and human and immunohistochemistry and PCR would not be similar; a different anti-NLRP3 antibody was used for hen and human immunohistochemistry and species-specific primers were used. There are several possible reasons that NLRP3 expression did not differ between cancerous and non-cancerous tissue: (i) a different NLR inflammasome (e.g., NLRP1 or NLRC4) is involved in IL18 production [37], or (ii) low-level NLRP3 expression leads to caspase-1 activation without significant synthesis [32]. Increased expression of NLRP1 occurs in breast cancer, and its transfection into cell lines promotes proliferation [38]. NLRP1, but not NLRP3, promotes melanoma (42). Although an NLRP1-like gene occurs in the hen [39], it was not possible to evaluate NLRP1 expression at this time since appropriate reagents are currently not available.

In response to specific stimuli, NLR expression is thought to be regulated in two steps. There are also canonical and non-canonical activation pathways. Canonical activation of the NLR inflammasome in response to a stimulus involves transcription and then oligomerization which proceed independently [40, 41]. Non-canonical NLR activation also involves transcription and oligomerization [40]. The primary caspase in canonical and non-canonical activation of IL18 production differs; canonical activation involves caspase-1 while non-canonical activation proceeds through caspase-11 (mouse)/caspase-4,5 (human) [41]. Factors and mechanisms in priming and activation remain to be clarified for most inflammasomes including NLRP3. For example, contrary to the initial models based on macrophage studies, in which the activated NLRP3 inflammasome is an oligomer of NLRP3, Salmonella simultaneously activates two NLRs, NLRP3 and NLRC4, and both are required in the same inflammasome oligomer to stimulate IL-1β processing [42]. In the current study, caspase-1 expression was significantly elevated while caspase-11 (non-canonical NLRP3 activation pathway) and caspase-8 (apoptosis pathway) did not change, suggesting activation of the canonical NLR pyroptosis pathway.

The aryl hydrocarbon receptor (AHR) is a ligand-activated transcription factor that regulates biological responses to aryl hydrocarbons, such as dioxin and dioxin-like compounds. It is present in various species and controls xenobiotic-metabolizing enzymes such as cytochrome P450s, principally CYP 1A1 [43]. AHR also has a role in immune function including, for example, in the generation of regulatory T cells, and control of the balance between Treg and Th17 cell differentiation [44]. AHR signaling has a critical function in carcinogenesis; AHR null mice lacking AHR developed liver tumors more frequently after exposure to a hepatic carcinogen (diethylnitrosamine), compared to wild type mice [45].

AHR is expressed in the human ovary and its expression does not vary during the menstrual cycle or with age [46]. Birds, including chickens, also express AHR [47, 48]. AHR has a role in follicle development and steroid hormone production since there are fewer advanced follicles and lower sex steroid production in AHR knockout mice [46].

AHR is constitutively activated in many cancers [49]. In human ovarian cancer, there is one report that AHR mRNA was not changed in primary tissue [46], while another study found that AHR expression was elevated in ovarian cancer cell lines [19]. We found that AHR expression was greater in human and chicken ovarian cancer.

AHRR represses AHR and may act as a tumor suppressor; reduced expression was reported in malignant ovarian tissue, and it is hypermethylated in OvCa [20]. AHRR is conserved in the chicken relative to the negative regulation of AHR and ERα activities, but functional mechanisms may differ among mammalian and fish homologs [48]. We found AHRR was elevated in human and chicken OvCa although it was only significant in the human tumors.

## Conclusions

Collectively, the data show that inflammasome components were higher in both hen and human OVCA. Inflammasome expression appears in immune cells and tumor cells. The specific NLR sensor remains to be determined since NLRP3 did not differ in ovarian tumors and normal ovary. Also, AHR expression increased similarly in hen and human tumors. Immune reactions may differ during the development and progression of a tumor and it is possible that additional or different reactions may be a response to a tumor. While the mechanisms involved in inflammasome effects on tumorigenesis remain to be determined, this study sets a platform for examining the relationship between tumor initiation and early inflammatory events in ovarian cancer development. Furthermore, the chicken model has potential use for pre-clinical testing of therapeutics targeting inflammasomes.

## Supporting information

S1 Table Demographic Data for Ovarian Cancer Patients. Tissue for mRNA was obtained at Rush University Medical Center (RUMC) (Chicago, IL). The pathologist provided the tumor type and stage. For IHC, tissue arrays were obtained from US BioMax, Inc. (Derwood, MD). Demographic and pathology data were provided in the specification sheet with each slide array (catalogue numbers provided in the Methods). Cancer adjacent normal ovary tissue was 1.5 cm away from the tumor.
(DOCX)

S2 Table Antibodies for Immunohistochemistry (IHC). Antibodies produced in the mouse or rat were monoclonal (MAB). Polyclonal antibodies were produced in goat or rabbit in response to recombinant peptides and were affinity purified. Catalogue number = cat #.
(DOCX)

S3 Table PCR primers. Primers were based on genes from the NCBI database. Chicken primers were orthologues of human genes. Oligoperfect Designer software (Invitrogen; Carlsbad, CA) was used to design each primer, and the endogenous actin control.
(DOCX)
