# Supplementary material for: Inflammasome expression is higher in ovarian tumors than in normal ovary
Source: PLoS One. 2020 Jan 10;15(1):e0227081. doi: 10.1371/journal.pone.0227081 (PMC6953783; doi:10.1371/journal.pone.0227081)
Supplement: S1 Table — Tissue for mRNA was obtained at Rush University Medical Center (RUMC) (Chicago, IL). The pathologist provided the tumor type and stage. For IHC, tissue arrays were obtained from US BioMax, Inc. (Derwood, MD). Demographic and pathology data were provided in the specification sheet with each slide array (catalogue numbers provided in the Methods). Cancer adjacent normal ovary tissue was 1.5 cm away from the tumor. (DOCX) [file pone.0227081.s001.docx]

**S1 Table.** **Demographic Data for Ovarian Cancer Patients**

| **USE** | **SITE** | **AGE** | **STAGE** | **GRADE** | **PATHOLOGY** | **TYPE** |
| --- | --- | --- | --- | --- | --- | --- |
| mRNA | RUMC | 72 | I | 1A | serous | malignant |
| mRNA | RUMC | 53 | I | 2A | clear cell | malignant |
| mRNA | RUMC | 80 | II | 3 | serous | malignant |
| mRNA | RUMC | 54 | IIA | 3 | serous | malignant |
| mRNA | RUMC | 61 | III | 3 | papillary serous | malignant |
| mRNA | RUMC | 50 | III | 2 | endometroid | malignant |
| mRNA | RUMC | 57 | IV | 3 | serous/endometrioid | malignant |
| mRNA | RUMC | 60 | IV | 3 | papillary serous | malignant |
| IHC | USBioMax | 40 | IIIc | 2–3 | Endometrioid carcinoma | Malignant |
| IHC | USBioMax | 40 | IIIc | 2–3 | Endometrioid carcinoma | Malignant |
| IHC | USBioMax | 39 | Ia | 3 | Serous adenocarcinoma | Malignant |
| IHC | USBioMax | 39 | Ia | 3 | Serous adenocarcinoma | Malignant |
| IHC | USBioMax | 41 | IIIc | 2–3 | Endometrioid carcinoma | Malignant |
| IHC | USBioMax | 52 | Ia | 2 | Mucinous adenocarcinoma | Malignant |
| IHC | USBioMax | 41 | Ia | 2 | Clear cell carcinoma | Malignant |
| IHC | USBioMax | 26 | Ic | 1 | Serous papillary adenocarcinoma | Malignant |
| IHC | USBioMax | 43 | I | 2 | Serous papillary adenocarcinoma | Malignant |
| IHC | USBioMax | 52 | Ib | 2 | Serous papillary adenocarcinoma | Malignant |
| IHC | USBioMax | 43 | I | 2 | Serous papillary adenocarcinoma | Malignant |
| IHC | USBioMax | 40 | IIIc | 2 | Endometrioid adenocarcinoma | Malignant |
| IHC | USBioMax | 42 | Ib | 1–2 | Mucinous adenocarcinoma | Malignant |
| IHC | USBioMax | 38 | – | – | Cancer adjacent ovary tissue | Adjacent Normal NNormal |
| IHC | USBioMax | 32 | – | – | Cancer adjacent ovary tissue | Adjacent Normal |
| IHC | USBioMax | 48 | – | – | Cancer adjacent ovary tissue | Adjacent Normal |
| IHC | USBioMax | 18 | – | – | Normal ovary tissue | Normal |
| IHC | USBioMax | 19 | – | – | Normal ovary tissue | Normal |
| IHC | USBioMax | 34 | – | –  – | Normal ovary tissue | Normal |
| IHC | USBioMax | 19 | – | –  – | Normal ovary tissue | Normal |
