# Supplementary material for: Inflammasome expression is higher in ovarian tumors than in normal ovary
Source: PLoS One. 2020 Jan 10;15(1):e0227081. doi: 10.1371/journal.pone.0227081 (PMC6953783; doi:10.1371/journal.pone.0227081)
Supplement: S2 Table — Antibodies produced in the mouse or rat were monoclonal (MAB). Polyclonal antibodies were produced in goat or rabbit in response to recombinant peptides and were affinity purified. Catalogue number = cat #. (DOCX) [file pone.0227081.s002.docx]

**S2 Table. Antibodies for Immunohistochemistry (IHC)**

| **ANTIGEN** | **SPECIES REACTIVITY** | **PRODUCED IN** | **VENDOR (cat #)** |
| --- | --- | --- | --- |
| CASPASE 1 | human | goat | R and D Systems (AF6215) |
| IL1β | chicken | rabbit | BioRad AbD Serotec (AHP941Z) |
| IL1β | human | mouse Mab | R and D Systems (MAB601) |
| IL18 | chicken | rabbit | MyBioSource (MBS2033795) |
| IL18 | human | goat | AbCam (ab 106939) |
| NLRP3 | human | rat Mab | R and D Systems (MAB 7578) |
| EpCAM | chicken | rabbit | MyBioSource (MBS2027145) |
