# Supplementary material for: Inflammasome expression is higher in ovarian tumors than in normal ovary
Source: PLoS One. 2020 Jan 10;15(1):e0227081. doi: 10.1371/journal.pone.0227081 (PMC6953783; doi:10.1371/journal.pone.0227081)
Supplement: S3 Table — Primers were based on genes from the NCBI database. Chicken primers were orthologues of human genes. Oligoperfect Designer software (Invitrogen; Carlsbad, CA) was used to design each primer, and the endogenous actin control. (DOCX) [file pone.0227081.s003.docx]

**S3 Table. PCR primers**

| **Species** | **Gene Name** | **Accession number** | **Forward primer** | **Reverse primer** |
| --- | --- | --- | --- | --- |
| chicken | NLRP3 | XM_001233261.3 | ATGAACCAGAGGGACCTTGC | CCTGGCTATGGTCAGGTTGG |
| chicken | CASP1 | XM_003642384.2 | TCTTCCCACAAGAAGTGGCT | AGCTCGTCCAGGAGATTGGA |
| chicken | CASP8 | NM_204592.2 | TCCTACAGAAGCCCAAGCCA | TGTCAATCTTGCTGCTCACCT |
| chicken | CASP11 | NW_003763474,1 | CCCCCACCATCTCAACAAGT | GTCCCTGAACAGTTCCCACA |
| chicken | IL1β | NM_204524.1 | GGGCATCAAGGGCTACAA | CTGTCCAGGCGGTAGAAGAT |
| chicken | IL18 | NM_204608 | AGTTGCTTGTGGTTCGTCCA | TCTACCTGGACGCTGAATGC |
| chicken | AHR | NM_204118.2 | TCCCATGATGGCAAATCAGCA | AGGATCTGGGCTCTGTTGGA |
| chicken | AHRR | NM_001201387.1 | GGGCCATGAATCCACCTCAG | GCAGCGAACTCGACAGATGA |
| chicken | CD3d | NM_205512 | TGATCCCAGAGGCACCTACA | ATCTGCGACCACAATCCCTG |
| chicken | CD3e | NM_206904.1 | CAGATCCGGCACTGGGAGA | TCCAGCTCCTCACAGTTTGC |
| chicken | CD4 | NM_204649.1 | CAGTGGAACCTGGATGTGTC | AGACGCCATGACAAGATTGC |
| chicken | CD8a | NM_205235.1 | GATTTTCATCTGGGCTCCCCT | CCTGCATCGTCGTCTTCTGG |
| chicken | CD8b | NM_205247.2 | CTACTACTGCTGCACCATCCA | GTTTCTTGGTGAGTGGGACC |
| chicken | CD45 | NM_204417.2 | CCTTCCTACAAGGGATGGCG | TTCACTCCTGATCGGCACTC |
| chicken | chB6 | NM_205182.1 | ACTGTGCCTGGGGAATTTGT | CGGGTACTGCAGAAGGTCTC |
| chicken | MRC1L-B | NM_001319013.1 | ACGACTTGATAGCAGCTGGG | TTTGCCATTCCTGCCTTGGA |
| chicken | EpCam | NM_001012564 | ACTCCTCTGAACGCTGAGTCT | GGGGTTTTCATACACAACACCA |
| chicken | PCNA | NM_204170.2 | CCATGGGCGTCAACCTAAAC | GCCAACGTATCCGCATTGTC |
| chicken | WT1 | NM_205216.1 | CTGAAACGGCACCAAAGACG | ACCTGTATGAGTCCTGGTATGA |
| chicken | MRC1L-B | NM_001319013.1 | ACGACTTGATAGCAGCTGGG | TTTGCCATTCCTGCCTTGGA |
| chicken | ACTIN | NM_205518.1 | TGGCAATGAGAGGTTCAGGT | ATGCCAGGGTACATTGTG GT |
| human | NLRP3 | XM_001233261.3 | ATGAACCAGAGGGACTTGC | CCTGGCTATGGTCAGGTTGG |
| human | CASP1 | XM_003642384.2 | ACTTCCACTTCGGATGGCTG | CTTCTGTGGGGCTTCTCCAG |
| human | IL1β | NM_204524.1 | GGGCATCAAGGGCTACAA | CTGTCCAGGCGGTAGAAGAT |
| human | IL18 | NM_204608 | AGTTGCTTGTGGTTCGTCCA | TCTACCTGGACGCTGAATGC |
